# Treating Acute Toxo-Metabolic Encephalopathy With Continuous Renal Replacement Therapy: A Case Report of Ifosfamide Neurotoxicity

**DOI:** 10.7759/cureus.103244

**Published:** 2026-02-08

**Authors:** Bilal Shahzad Azam Khan, Sumayya Din Bashir

**Affiliations:** 1 Department of Internal Medicine, Shaukat Khanum Memorial Cancer Hospital and Research Centre, Lahore, PAK

**Keywords:** acute kidney injury, acute toxometabolic encephalopathy, continuous renal replacement therapy, ifosfamide, volume of distribution

## Abstract

Acute toxometabolic encephalopathy, which encompasses delirium and the acute confusional state, is a condition of global cerebral dysfunction in the absence of primary structural brain disease. Ifosfamide is an alkylating agent used in chemotherapy for the treatment of hematological and various solid tumors and is associated with dose-dependent central nervous system toxicity. We report a case of a patient who developed severe neurological symptoms and encephalopathy after receiving Ifosfamide, a chemotherapy agent. Despite initial management with intravenous methylene blue, the patient continued to deteriorate. Initiation of continuous venovenous hemodialysis 14 hours after symptom onset resulted in rapid and complete recovery.

## Introduction

Ifosfamide is widely used in combination chemotherapy regimens for hematologic malignancies and various solid tumors [1]. Its use is complicated by the potential for central nervous system (CNS) toxicity in up to 16% of patients [2] and is thought to result from the accumulation of the metabolite, chloroacetaldehyde, which depletes neuronal glutathione and disrupts mitochondrial function. Management of neurotoxicity involves prompt drug discontinuation, intravenous fluids, administration of methylene blue, and electroencephalographic monitoring [3]. We present a case of severe ifosfamide-induced encephalopathy treated with extracorporeal clearance of ifosfamide metabolites with continuous venovenous hemodialysis (CVVHD), leading to rapid recovery in a patient with relapsed high-grade non-Hodgkin lymphoma.

## Case presentation

**Figure 1:**
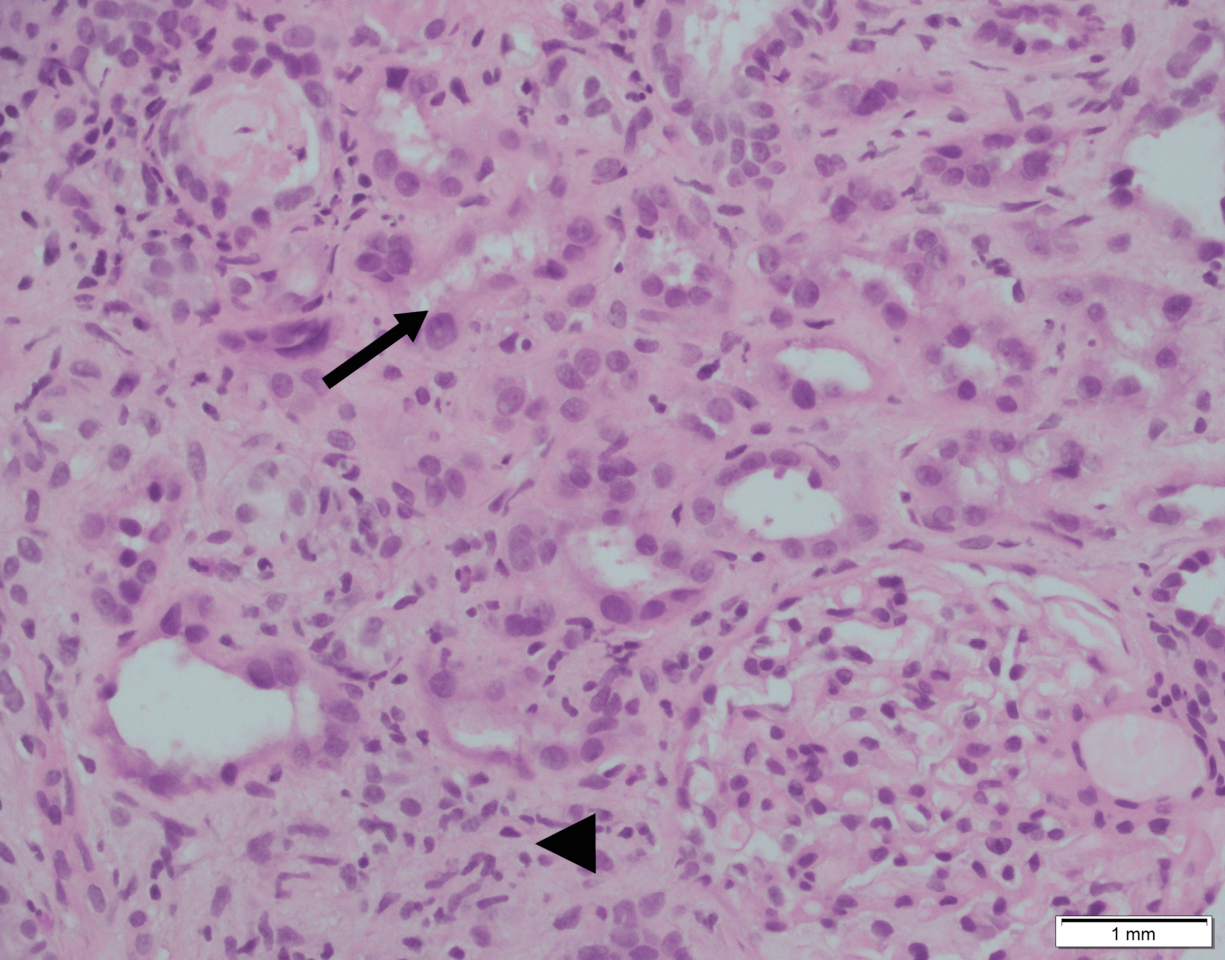
Renal biopsy on light microscopy demonstrating tubular injury (arrow) and interstitial fibrosis (arrowhead) (hematoxylin and eosin stain, magnification ×400).

We present a case report of a 39-year-old male undergoing treatment at Shaukat Khanum Memorial Cancer Hospital, Lahore, for relapsed high-grade non-Hodgkin lymphoma (NHL). His medical history included a hepatitis C virus infection, which was treated in February 2025, and a right-sided ureteric stone, which was managed with lithoclast fragmentation and right ureteric stenting in January 2025. As initial treatment, he received chemotherapy from June to September 2024, but unfortunately, he relapsed. He was restarted on chemotherapy in March 2025. In April 2025, he was admitted for neutropenic sepsis and stage III acute kidney injury (AKI). Serum creatinine rose from a baseline of 0.82 mg/dL to 6.1 mg/dL, and he required continuous renal replacement therapy (CRRT) during this admission. Renal function improved after recovery from neutropenic sepsis, and he was weaned off dialysis, although he was left with chronic kidney disease. Serum creatinine at discharge was 4.6 mg/dL. A renal biopsy after discharge confirmed severe acute tubular injury, moderate acute-on-chronic interstitial nephritis, and global glomerulosclerosis. Moderate interstitial fibrosis and tubular atrophy suggested permanent renal parenchymal damage (Figure 1).

In May 2025, he was admitted for inpatient chemotherapy with rituximab, ifosfamide, etoposide, and epirubicin. His pre-chemotherapy serum creatinine was 4.23 mg/dL, with a creatinine clearance of 31 mL/min by the Cockcroft-Gault equation and an estimated glomerular filtration rate of 18 mL/min/1.73 m² by the Chronic Kidney Disease Epidemiology Collaboration (CKD-EPI) 2021 equation. This prompted his oncologist to reduce the dose of ifosfamide by 50% and etoposide by 25%. Given his high risk for tumor lysis syndrome, prophylactic rasburicase was also administered. Near the completion of his ifosfamide infusion, the patient developed an acute confusional state, associated with generalized muscular twitching and jerky limb movements. He subsequently deteriorated and became unresponsive with a drop in his Glasgow Coma Scale (GCS) to 9/15 from a pre-chemotherapy GCS of 15/15. Ifosfamide neurotoxicity was suspected, and intravenous methylene blue, along with intravenous fluids, was initiated. Laboratory work showed no significant changes from his baseline renal function. Despite continuing these measures, no improvement in his mental status was observed over the next 14 hours. On the Naranjo Adverse Drug Reaction Probability Scale, a score of six suggested a probable adverse drug reaction (Table 1) [4].

**Table 1:** Adapted from Naranjo Adverse Drug Reaction Probability Scale [4]

Adverse Drug Reaction Probability Scale	Input
Are there previous conclusive reports on this reaction?	Yes
Did the adverse event appear after the suspected drug was given?	Yes
Did the adverse reaction improve when the drug was discontinued or a specific antagonist was administered?	Yes
Did the adverse event reappear when the suspected drug was re-administered?	Do Not Know
Are there alternative possible causes for the reaction?	No
Did the adverse reaction reappear when placebo was given?	Do Not Know
Was the drug detected in the blood or other body fluids in concentrations known to be toxic?	Do Not Know
Was the reaction worsened upon increasing the dose or decreased on reducing the dose?	Do Not Know
Did the patient have a similar reaction to the suspected drug or a similar drug in the past?	No
Was the adverse reaction confirmed by any other objective evidence?	No
Final Score	+ 6
Interpretation	Probable. The reaction occurred after the drug was given, and possibly followed a pattern that matches a drug reaction that is reasonably explained as an adverse drug reaction

Nephrology consultation was sought, and a trial of Continuous Venovenous Hemodialysis (CVVHD), a form of CRRT, was recommended to help remove Ifosfamide toxic metabolites. The patient was transferred to the intensive care unit, dialysis access was secured, and CVVHD was initiated with a blood flow rate of 200 mL/min and a dialysate flow rate of 2000 mL/h. The patient’s mental status and muscular twitching rapidly improved, resolving completely within 12 hours of starting CVVHD. Given the significant improvement in his neurological symptoms, CVVHD was discontinued, and he was stepped down to the floor, where intravenous fluids were continued. Blood urea nitrogen improved from a pre-dialysis value of 53mg/dL to 29 mg/dL at discontinuation, which shows the effectiveness of clearance with CRRT. An electroencephalogram and brain imaging were planned, but were no longer deemed necessary given the improvement in symptoms. His oncologist decided to switch to an alternative chemotherapy regimen in view of the serious adverse effects observed with Ifosfamide.

## Discussion

Ifosfamide is an oxazaphosphorine alkylating agent and an isomer of cyclophosphamide [1]. This hydrophilic prodrug is metabolized in the liver by the cytochrome P450 system into phosphoramide mustard, 4-hydroxy-ifosfamide, and acrolein. These metabolites induce cell damage by forming interstrand or intrastrand crosslinks, leading to apoptosis of the affected cells. The active metabolites also upregulate reactive oxygen species (ROS), resulting in irreparable DNA damage and cessation of protein synthesis [5]. In addition to these metabolites, dechloroethylation produces chloroacetaldehyde, which is possibly linked to the development of neurotoxicity in a dose-dependent manner. The volume of distribution (Vd) of ifosfamide approximates total body water, with minimal tissue binding [6]. Its half-life is approximately 15 hours at high doses (3800-5000 mg/m²) and seven hours at lower doses (1800-2400 mg/m²). Elimination occurs via renal filtration, and dosage adjustments are required based on renal function. Dose reductions of 80%, 75%, and 70% of the standard dose are recommended for creatinine clearances (CrCl) of 46-60 mL/min, 31-45 mL/min, and below 30 mL/min, respectively [7]. In our patient, a higher dose and poor renal function might have been major risk factors for neurotoxicity. 

Acute toxometabolic encephalopathy (TME), which encompasses delirium and acute confusional states, is a condition of global cerebral dysfunction in the absence of primary structural brain disease [8]. Ifosfamide-induced encephalopathy is a clinical diagnosis with a wide spectrum of signs and symptoms, including impaired consciousness, lethargy, somnolence, confusion, hallucinations, delusions, irritability, anxiety, disorientation, weakness, seizures, movement disorders, extrapyramidal symptoms, tremors, and coma [9]. According to the National Cancer Institute, there are five severity grades of encephalopathy [10], with most patients who develop ifosfamide-induced encephalopathy exhibiting grade one or two symptoms. A minority develops grade three or four symptoms. Most cases resolve spontaneously, but rare cases of grade four encephalopathy can result in permanent brain damage or death [3]. Risk factors include low serum albumin, poor performance status, and renal insufficiency [2,11]. Based on a literature search, ours is one of the few reported cases in which CVVHD was used to treat ifosfamide-induced neurotoxicity (Table 2).

**Table 2:** Summary of previous published case reports of ifosfamide neurotoxicity treated with hemodialysis and CRRT in comparison to the current case CVVHD: continuous veno-venous hemodialysis; CRRT: continuous renal replacement therapy; mg/m²: milligrams per square meter of body surface area; GCS: Glasgow coma scale

Study	Diagnoses	Age/Gender	Ifosfamide dose	Chemotherapy regimen	Neurological symptoms	Dialytic Modality	Outcome
Current Patient	Relapsed Non-Hodgkin Lymphoma	39-year-old male	1500mg/m2	Rituximab, Ifosfamide, Etoposide, Epirubicin	Decreased GCS, muscular jerks and twitching	CVVHD	Mental status improved and muscular jerks resolved completely
Yeo et al. 2016 [12]	Relapsed Neuroblastoma	11-year-old female	2000mg/m2	Ifosfamide, Carboplatin, Etoposide	Decreased GCS, loss of gag reflex	CVVHD	Improvement in neurological symptoms
Nishimura et al. 2014 [13]	Metastatic osteosarcoma	15-year-old Female	3000mg/m2	Ifosfamide and Etoposide	Confusion, hallucinations, agitation, and coma	Hemodialysis & Hemofiltration	Reversal of symptoms
Mohammad A Cherry et al. 2012 [14]	Relapse Non-Hodgkin Lymphoma	67-year-old male	5000mg/m2	Rituximab, Ifosfamide, Carboplatin, Etoposide	Confusion followed by coma	CVVHD	Resolution of symptoms
Fiedler R et al. 2001 [15]	Relapsed Non-Hodgkin Lymphoma	38-year-old male	5000mg/m2	Ifosfamide, Carboplatin, Etoposide	Confusion and muscular weakness	Hemodialysis & Hemofiltration	Resolution of symptoms

Ifosfamide-induced encephalopathy may develop anywhere from 2 to 96 hours after administration and typically resolves spontaneously within 48-72 hours after discontinuation, although it may persist longer in some cases [6]. Chloroacetaldehyde is the most likely neurotoxic metabolite of ifosfamide. Its neurotoxic effects have been postulated to result from direct neuronal toxicity, depletion of CNS glutathione, and inhibition of mitochondrial oxidative phosphorylation, leading to impaired fatty acid metabolism [16]. Elevated blood levels of chloroacetaldehyde have been documented in patients with ifosfamide-induced neurotoxicity, suggesting a dose-dependent toxicity relationship. Peak levels of this metabolite develop approximately four hours after ifosfamide infusion [17].

Ifosfamide-induced neurotoxicity is usually reversible by discontinuing the ifosfamide infusion and initiating intravenous hydration, although additional interventions may be required. The most commonly used pharmacological treatment is methylene blue, an alternative electron acceptor that facilitates detoxification of neurotoxic metabolites. Its efficacy for treatment and prophylaxis has been studied extensively, with variable results in cases of severe toxicity [18].

With a molecular weight of 78.5 daltons, chloroacetaldehyde is classified as a small molecule. Due to minimal binding and rapid distribution equilibrium between erythrocytes and plasma, it can be efficiently removed by continuous venovenous hemodialysis (CVVHD), which primarily relies on diffusion for clearance. Levels of chloroacetaldehyde have been reported to decrease by a mean of 77.2% following dialysis [17]. This may explain how prompt initiation of CRRT in our patient led to rapid improvement in neurological status.

## Conclusions

This case highlights the importance of multidisciplinary coordination among oncologists, nephrologists, and intensivists in improving patient outcomes. Continuous venovenous hemodialysis (CVVHD) may have a role in treating severe toxometabolic encephalopathy secondary to ifosfamide. Keeping in mind limitations like single-case design and the possibility of spontaneous recovery, a larger multi-center study of registry data and standardized methylene blue protocols is needed to clarify the role of early continuous renal replacement therapy (CRRT) in managing severe ifosfamide-induced encephalopathy.
